# Dulaglutide as a Bridging Therapy Before Insulin for Diabetes Following Pancreatectomy on Congenital Hyperinsulinism

**DOI:** 10.1210/jcemcr/luaf281

**Published:** 2025-12-03

**Authors:** Sakura Motegi, Masanori Adachi, Keiko Nagahara, Ayako Ochi, Tatsuyuki Ishida, Katsumi Mizuno

**Affiliations:** Department of Pediatrics, Showa Medical University, Tokyo 142-8666, Japan; Department of Pediatrics, Showa Medical University, Tokyo 142-8666, Japan; Department of Pediatrics, Showa Medical University, Tokyo 142-8666, Japan; Department of Pediatrics, Showa Medical University, Tokyo 142-8666, Japan; Department of Pediatrics, Showa Medical University, Tokyo 142-8666, Japan; Department of Pediatrics, Showa Medical University, Tokyo 142-8666, Japan

**Keywords:** β-cell function, congenital hyperinsulinism, dulaglutide, hyperinsulinism and postpancreatectomy diabetes, glucagon-like peptide-1 receptor agonist

## Abstract

Hyperinsulinism and postpancreatectomy diabetes (CHI/PPD) refers to diabetes following pancreatectomy to treat hypoglycemia due to congenital hyperinsulinism. Insulin therapy in CHI/PPD may sometimes be challenging because of glucagon deficiency and the dysfunction of the remaining β-cells. In a 16-year-old male patient with CHI/PPD and severe hypoglycemic brain damage, a dulaglutide trial was planned, because some biochemical indices suggested that he was not completely insulin-dependent. Other than transient appetite loss, no adverse events were observed. The patient's hemoglobin A1c level promptly decreased from 6.7% (SI: 49.7 mmol/mol) [reference range, 4.6-6.2% (SI: 26.8-44.3 mmol/mol)] to 5.7%, with temporary, but not persistent, improvement in endogenous insulin secretion. He has been free from exogenous insulin for more than 4 years under dulaglutide. In conclusion, in selected patients with CHI/PPD and reserved β-cell function, dulaglutide may serve as a bridging therapy before insulin introduction.

## Introduction

Hyperinsulinism and postpancreatectomy diabetes (CHI/PPD) refers to diabetes that develops following subtotal (≤95%) or near-total (95% to 98%) pancreatectomy performed to treat congenital hyperinsulinism (CHI) [1, 2]. CHI is a genetic disorder characterized by insulin hypersecretion and resultant hypoglycemia that often develops during infancy. According to the pathology, CHI is classified into either diffuse or focal form. Currently, medical treatment using a combination of a transfusion with a high glucose infusion rate, diet therapy, steroids, and diazoxide is the mainstay for treatment because spontaneous resolution occurs frequently [3, 4]. However, decades prior, prompt pancreatectomy was encouraged, emphasizing the importance of avoiding intellectual damage from repetitive hypoglycemia [3, 5, 6]. To eradicate hypoglycemia, pancreatectomy ≥95% has been performed. As a price of cure of hypoglycemia, CHI/PPD occurs frequently, immediately postsurgery or, more commonly, with a latency of several years. In 1 report, 43 out of 45 patients developed CHI/PPD within 11 years of undergoing ≥95% pancreatectomy [7]. Another study demonstrated that all 58 patients who underwent subtotal pancreatectomy developed elevated blood glucose (BS) levels at 14 years after the procedure [8].

Achieving adequate insulin treatment for patients with CHI/PPD remains challenging [1, 2, 7-9]. Patients receiving exogenous insulin may easily develop hypoglycemia due to glucagon deficiency. In addition, irregular insulin secretion from the remaining β-cells may cause unexpected BS fluctuation. Therefore, a bihormonal (insulin plus glucagon) bionic pancreas utilizing a continuous glucose monitoring device has been proposed as a promising treatment option [2].

Our patient, whose pancreas was subtotally resected at 7 months of age, developed CHI/PPD 16 years later. Because he had severe intellectual problems due to hypoglycemic brain damage, appropriate insulin therapy seemed even more difficult. Because biochemical studies indicated that the patient was not completely insulin-dependent, we introduced dulaglutide, a long-acting glucagon-like peptide-1 receptor agonist (GLP1RA). Here we report on the more than 4-year course of treatment.

## Case Presentation

The patient was born at term via vaginal delivery and weighed 3685 g. Asphyxia was absent. At 12 hours of life, he was transferred to a local hospital because of respiratory distress. He was diagnosed with CHI based on profound hypoglycemia of BS 19 mg/dL (SI: 1.05 mmol/L) [reference range, 70-109 mg/dL (SI: 3.9-6.1 mmol/L)] with nonsuppressed insulin level: the ratio of immunoreactive insulin (µIU/mL) to BS (mg/dL) was 2.2. Hyperammonemia of 130 µg/dL (SI: 76.3 µmol/L) [reference range, 15-80 µg/dL (SI: 8.8-47.0 µmol/L)] was also observed. However, genetic analysis failed to detect any pathogenic variants in the candidate genes for CHI, including *GLUD1*. He was mainly treated with transfusion with glucose infusion rate up to 20 mg/kg/min, combined with frequent feeding of a leucine-free formula. Diazoxide was attempted but withdrawn because of vomiting, edema, and tremors. Systemic steroid administration also failed. Accordingly, a subtotal pancreatectomy was performed at 7 months of age, which led to the resolution of hypoglycemia. The resected pancreatic tissue weighed 7 g, and the pathological investigation was consistent with a diagnosis of diffuse CHI.

Because of family relocation, the patient was referred to Showa Medical University Hospital at 12 years of age. He showed no signs of hypoglycemia. He had an intellectual disability and motor paralysis. He spoke only nonverbal words and used a wheelchair or crawled to move. Because the patient has difficulty in chewing and swallowing, his food has been cut into easy-to-eat small pieces since childhood. The limited capacity for oral feeding and the resultant long-lasting undernutrition resulted in significant short stature and thinness. He needed to take valproate because he developed generalized convulsions at 12 years of age, which were unrelated to hypoglycemia, and he was diagnosed with epilepsy. The hemoglobin A1c (HbA1c) level began to increase after 14 years old, which synchronized with pubertal progression.

## Diagnostic Assessment

**Figure 1.**
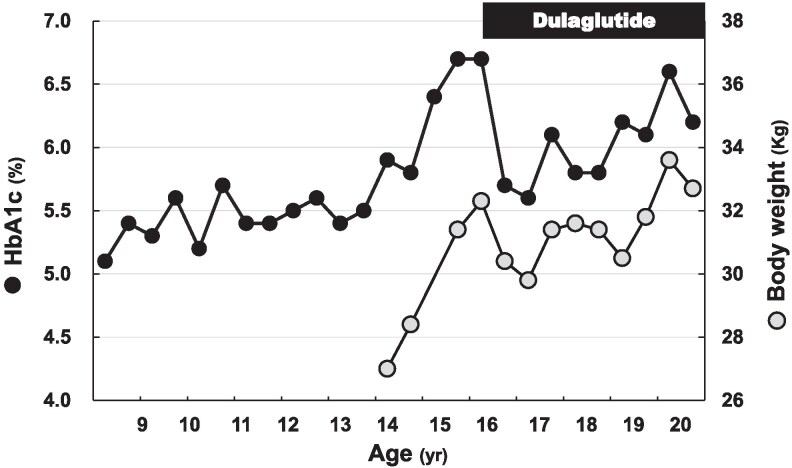
Time course of hemoglobin A1c level and body weight.

**Figure 2.**
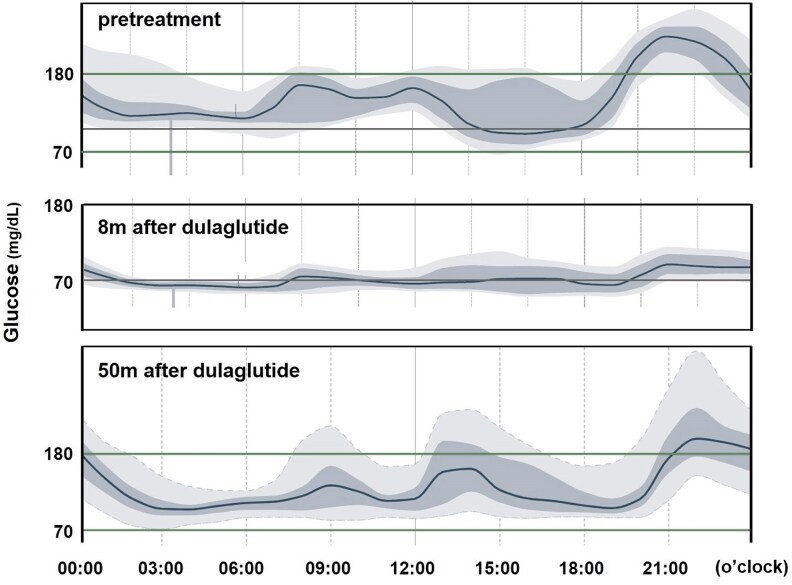
Blood glucose profiles obtained by continuous glucose monitoring, demonstrating 95%, 75%, 50%, 25%, and 5% of blood glucose levels. Upper panel: pretreatment; middle panel: 8 months after dulaglutide treatment; lower panel: 50 months after dulaglutide treatment.

At 16 years of age, because the patient's HbA1c level rose to 6.7% (Fig. 1), a thorough evaluation of his glucose metabolism was planned to determine his treatment options. On admission, he was short [148.0 cm (−3.8SD)] and had a body weight (BW) of 31.4 kg with a body mass index (BMI) of 14.3 kg/m^2^ (−3.9SD). His heart rate was 78/min; blood pressure, 112/66 mmHg; and respiratory rate, 26/min. Physical examination was unremarkable except for thinness, and the patient showed adequate pubertal development with Tanner IV pubic hair. Complete blood cell counts and comprehensive metabolic panel, including thyroid function, were entirely normal. Ketonuria was absent. The results of the oral glucose tolerance test (OGTT) and IV glucagon stimulation test (GST) are shown in Table 1. He met the diagnostic criteria for diabetes in Japan [11]. Continuous glucose monitoring revealed postprandial hyperglycemia, which was most evident at night (Fig. 2).

**Table 1.** Test results before and after dulaglutide treatment

Pretreatment			50 m after dulaglutide		
Glucagon stimulation test			Glucagon stimulation test		
	Blood glucose	CPR		Blood glucose	CPR
0 minutes	106 mg/dL [5.9 mmol/L]	0.70 ng/mL [0.23 nmol/L]	0 minutes	83 mg/dL [4.6 mmol/L]	0.89 ng/mL [0.29 nmol/L]
6 minutes	128 mg/dL [7.1 mmol/L]	1.93 ng/mL [0.64 nmol/L]	6 minutes	96 mg/dL [5.3 mmol/L]	1.37 ng/mL [0.45 nmol/L]

Oral glucose tolerance test			Oral glucose tolerance test		
	Blood glucose	IRI		Blood glucose	IRI
0 minutes	106 mg/dL [5.9 mmol/L]	2.1 μIU/mL [14.6 pmol/L]	0 minutes	83 mg/dL [4.6 mmol/L]	2.1 μIU/mL [14.6 pmol/L]
30 minutes	144 mg/dL [8.0 mmol/L]	10.8 μIU/mL [75.0 pmol/L]	30 minutes	213 mg/dL [11.8 mmol/L]	24.2 μIU/mL [168 pmol/L]
60 minutes	142 mg/dL [7.9 mmol/L]	4.4 μIU/mL [30.6 pmol/L]	60 minutes	279 mg/dL [15.5 mmol/L]	44.9 μIU/mL [312 pmol/L]
120 minutes	308 mg/dL [17.1 mmol/L]	54.0 μIU/mL [375 pmol/L]	120 minutes	221 mg/dL [12.3 mmol/L]	49.5 μIU/mL [344 pmol/L]

Interpretation of glucagon stimulation test is shown in Table 2. In oral glucose tolerance test, blood glucose >200 mg/dL [SI: >11.1 mmol/L] is the criterion for diabetic pattern [10].

Abbreviations: CPR, C-peptide immunoreactivity; IRI, immunoreactive insulin.

As shown in Table 2, homeostatic model assessment of β-cell function was 17.6%, indicating impaired insulin secretion. However, C-peptide immunoreactivity (CPR)-based indices of endogenous insulin secretion showed mixed results: whereas C-peptide index [CPI; 100 × fasting CPR (ng/mL)/BS (mg/dL)] was 0.66 and indicated insulin requirement, others [fasting CPR, GST-stimulated CPR, and ΔCPR (0-6 minutes)] suggested that his β-cell function was still retained.

**Table 2.** Trends in biochemical indices of endogenous insulin secretion

	Pretreatment (16 years)	8 m after dulaglutide (17 years)	50 m after dulaglutide (20 years)	Significance of each index
IRI-based index				
HOMA-β	**17.6%**	63.0%	37.8%	<30% indicates decreased insulin secretion
CPR-based indices				
C-peptide index	**0.66**	1.08	1.07	<1.0 predicts insulin requirement in T2D [12]
Fasting CPR	0.70 ng/mL [0.23 nmol/L]	0.81 ng/mL [0.27 nmol/L]	0.89 ng/mL [0.29 nmol/L]	<0.5 ng/mL [<0.17 nmol/L] indicates insulin-dependency in T2D [10]
GST-stimulated CPR	1.93 ng/mL [0.64 nmol/L]	ND	1.37 ng/mL [0.45 nmol/L]	<1.0 ng/mL [<0.33 nmol/L] indicates insulin-dependency in T2D [10]
ΔCPR (0-6 minutes)	1.23 ng/m l [0.41 nmol/L]	ND	**0.48 ng/mL** **[0.16 nmol/L]**	<0.5 ng/mL [<0.17 nmol/L] indicates insulin-dependency in T2D [10]

Values below the threshold are in bold.

Abbreviations: CPR, C-peptide immunoreactivity; GST, IV glucagon stimulation test; HOMA-β, homeostatic model assessment of β-cell function; IRI, immunoreactive insulin; ND, no data; T2D, type 2 diabetes.

## Treatment

Collectively, we judged that the patient had overt diabetes with impaired insulin secretion but was not completely insulin-dependent. Although insulin therapy seemed ideal, it was problematic because of the possibility of unexpected BS fluctuations, as stated previously, and the difficulty in managing hypoglycemia. Metformin and thiazolidinedione seemed unsuitable because the patient was insulin-sensitive. We were reluctant to use sulfonylurea because it would cause hypoglycemia and may enhance β-cell loss [13]. Instead, dulaglutide therapy was planned to restore insulin secretion and for its possible antiapoptotic effects. With the approval of the Showa Medical University Research Ethics Review Board (permission number: 2102-T-00054), weekly administration of dulaglutide 0.75 mg was initiated.

## Outcome and Follow-up

During the initial several months under dulaglutide, the patient lost his appetite, and his BW decreased from 31.4 kg (BMI −4.1 SD) to 29.8 kg (BMI −5.0 SD), corresponding to 5.1% BW loss. However, he did not present with vomiting, diarrhea, or any signs of hypoglycemia. Thereafter, his appetite gradually returned to pretreatment status, with complete recovery of BW within a year. No other adverse events related to dulaglutide were observed.

After 3 months, the patient's HbA1c level decreased from 6.7% to 5.7% (Fig. 1). At 8 months, continuous glucose monitoring revealed suppressed BS in the fasting and postprandial states (Fig. 2). In addition, the OGTT showed a normal pattern (data not shown), and all available indices of endogenous insulin secretion improved (Table 2). However, at 50 months, the HbA1c level increased to 6.6%, prompting us to perform OGTT and GST again. At the time, he weighed 33.6 kg (BMI −3.2SD). Although ketonuria was still absent, the OGTT showed a diabetic pattern again (Table 1). Compared with those before treatment, some indices (homeostatic model assessment of β-cell function, CPI, and fasting CPR) improved, whereas others [GST-stimulated CPR and ΔCPR (0-6 minutes)] deteriorated (Table 2). Based on these findings, dietary carbohydrate intake was reduced, which resulted in a 0.9 kg BW decrease and HbA1c improvement to 6.2% in 2 months.

## Discussion

To the best of our knowledge, this is the first trial on GLP1RA in patients with CHI/PPD. In our patient, the BS-lowering effect of dulaglutide was observed soon after its introduction. In addition, the patient did not require insulin therapy and maintained acceptable HbA1c level for more than 4 years. Therefore, we argue that dulaglutide could delay the introduction of insulin.

We attributed this success to the preservation of endogenous insulin secretion. Whereas short-acting GLP1RAs suppress postprandial hyperglycemia mainly by delaying gastric emptying, long-acting GLP1RAs such as dulaglutide and liraglutide lower fasting and postprandial BS by enhancing insulin secretion and reducing glucagon secretion [14-16]. The BS- lowering effect of liraglutide among patients with type 2 diabetes (T2D) depends on residual β-cell function estimated by ΔCPR (0-6 minutes) or CPI [17, 18].

In addition to the restoration of insulin secretion, we anticipated that dulaglutide might exert an antiapoptotic effect on the remaining β-cells, as some experimental data have shown the antiapoptotic effects of GLP1RA [19, 20]. Moreover, in T2D, the early introduction of GLP1RA is believed to be beneficial in protecting β-cells from apoptosis [21]. Furthermore, the insulin-sparing effect of GLP1RA has been recently reported in adults with new-onset type 1 diabetes [22]. In contrast, enhanced apoptosis has been demonstrated in β-cells of CHI. This partly explains the spontaneous resolution of hypoglycemia and the later development of diabetes in patients with CHI, even in those who have not undergone pancreatectomy [23, 24]. Accordingly, patients with CHI following pancreatectomy, such as our patient, are at a particularly high risk of total insulin dependence.

However, contrary to our expectations, the improvement in the patient's glucose tolerance was transient, and GST-stimulated CPR and ΔCPR (0-6 minutes) worsened compared with those before treatment. These results were not consistent with the antiapoptotic effects of dulaglutide. Thus, we assumed that the patient would become insulin-dependent in near future. Whether the introduction of GLP1A soon after pancreatectomy would exert antiapoptotic effect is another issue that should be verified in future studies.

No comparative studies have been performed among the indices used here to evaluate β-cell function. So the significance of the discrepant results of these indices observed in the patient is unknown. The thresholds of the CPR-based indices were excerpted from the studies on T2D. CPI <1.0 was proposed as a predictor of insulin requirement in T2D [12]. Thresholds for fasting CPR (0.5 ng/mL), GST-stimulated CPR (1.0 ng/mL), and ΔCPR (0-6 minutes) (0.5 ng/mL) have been used to evaluate insulin dependence in Japanese patients with T2D [10]. Therefore, these thresholds may not directly apply to patients with CHI/PPD.

When expanding dulaglutide trials to other patients with CHI/PPD, several issues must be addressed. First, anorexia is a well-known class effect of GLP1RA [14, 15], which resulted in 5.1% weight loss in our patient. Accordingly, strict monitoring for hypoglycemia is needed, especially in the early phase. Second, in patients with limited β-cell capacity for insulin secretion, the possibility of treatment failure should always be considered, which may lead to serious complications such as ketoacidosis. Finally, patients, caregivers, and medical professionals should be aware that dulaglutide is a bridging therapy and that insulin treatment will eventually be necessary.

In conclusion, a patient with CHI/PPD and residual endogenous insulin secretion responded to dulaglutide and remained insulin-free for more than 4 years. Dulaglutide may be a therapeutic option as a bridging intervention for CHI/PPD.

## Learning Points

- CHI/PPD is a unique form of diabetes that occurs following surgical intervention for CHI.
- CHI/PPD almost inevitably occurs in patients with CHI who undergo subtotal pancreatectomy, necessitating insulin therapy.
- In a patient with CHI/PPD, dulaglutide temporarily improved endogenous insulin secretion and delayed the introduction of exogenous insulin for more than 4 years.
- For selected patients with CHI/PPD, especially those with reserved β-cell function, dulaglutide may serve as a bridging therapy before insulin treatment.

## Data Availability

The original data generated and analyzed in this case report are included in this published article.
